# Is Hypoalbuminemia a Predictor for Acute Kidney Injury after Coronary Bypass Grafting in Diabetes Mellitus Patients?

**DOI:** 10.21470/1678-9741-2018-0291

**Published:** 2019

**Authors:** Rezan Aksoy, Taylan Adademir, Ekrem Yilmaz, Deniz Cevirme, Mehmet Sengor, Cengiz Koksal, Murat Bulent Rabus

**Affiliations:** 1 Department of Cardiovascular Surgery, University of Health Sciences, Kartal Kosuyolu Heart Education and Research Hospital, Istanbul, Turkey.; 2 Department of Cardiovascular Surgery, Bezmialem Vakıf University, Medical Faculty, Istanbul, Turkey.

**Keywords:** Coronary Bypass, Diabetes Mellitus, Hypoalbuminemia

## Abstract

**Objective:**

Acute kidney injury (AKI) is one of the most important complications after coronary artery bypass grafting (CABG) procedure. Serum albumin, which is an acute phase reactant, is suggested to be associated with AKI development subsequent to various surgical procedures. In this study, we research the relation between preoperative serum albumin levels and postoperative AKI development in diabetes mellitus (DM) patients undergoing isolated CABG.

**Methods:**

We included a total of 634 diabetic patients undergoing CABG (60.5±9.1 years, 65.1% male) into this study, which was performed between September 2009 and January 2014 in a single center. The relation between preoperative serum albumin levels and postoperative AKI development was observed. AKI was evaluated and diagnosed using the Kidney Disease: Improving Global Outcomes (KDIGO) classification.

**Results:**

AKI was diagnosed in 230 (36.3%) patients. Multiple logistic regression analysis was performed to determine the independent predictors of AKI development. Proteinuria (odds ratio [OR] and 95% confidence interval [CI], 1.066 [1.002-1.135]; *P*=0.043) and low preoperative serum albumin levels (OR and 95% CI, 0.453 [0.216-0.947]; *P*=0.035) were found to be independent predictors of AKI. According to the receiver operating characteristic curve analysis, albumin level <3mg/dL (area under the curve: 0.621 [0.572-0.669], *P*<0.001) had 83% sensitivity and 10% specificity on predicting the development of AKI.

**Conclusion:**

We observed that a preoperative low serum albumin level was associated with postoperative AKI development in patients with DM who underwent isolated CABG procedure. We emphasize that this adjustable albumin level should be considered before the operation since it is an easy and clinically implementable management for the prevention of AKI development.

**Table:** 

Abbreviations, acronyms & symbols				
ACC	= Aortic cross-clamping		Hgb	= Hemoglobin
AKI	= Acute kidney injury		HT	= Hypertension
ANOVA	= Analysis of variance		ICU	= Intensive care unit
AUC	= Area under the curve		IQR	= Interquartile range
BMI	= Body mass index		KDIGO	= Kidney Disease: Improving Global Outcomes
CABG	= Coronary artery bypass grafting		LVEF	= Left ventricular ejection fraction
CI	= Confidence interval		MI	= Myocardial infarction
CPB	= Cardiopulmonary bypass		OR	= Odds ratio
CRP	= C-reactive protein		RDW	= Red cell distribution width
DM	= Diabetic mellitus		ROC	= Receiver operating characteristic
EDTA	= Ethylenediaminetetraacetic acid		SD	= Standard deviation
EF	= Ejection fraction		SCr	= Serum creatinine
GFR	= Glomerular filtration rate		SPSS	= Statistical Package for Social Sciences

## INTRODUCTION

Twenty to 30% of all patients undergoing coronary artery bypass grafting (CABG) are diabetic^[1]^. Patients with type 2 diabetes mellitus (DM) have been reported to show high morbidity and mortality rates following CABG operations^[1]^. Type 2 DM is reported to increase postoperative acute kidney injury (AKI) development rates in patients undergoing CABG surgery^[2,3]^. AKI, which is not rarely seen after cardiac surgery, is associated with increased morbidity and mortality rates. AKI subsequent to cardiac surgery is diagnosed in 5-30% of the patients and renal replacement therapy is required in 1-2% of them^[4]^. And AKI subsequent to CABG is also associated with longer intensive care unit (ICU) and in-hospital stays and increased rates of hemodialysis requirement and chronic renal failure^[5]^. Serum albumin, which is a plasma protein that has an important role on the regulation of plasma oncotic pressure, is also is an acute-phase reactant. The normal range of albumin in serum is 3-5 g/dL^[6]^. In many studies, hypoalbuminemia subsequent to cardiac surgical procedures was found to be associated with increased rates of mortality and morbidity^[7,8]^. There are also studies showing that hypoalbuminemia is associated with AKI development after various surgical procedures^[6]^. In this study, we aimed to research the relation between preoperative serum albumin levels and postoperative AKI development in selected patients with DM undergoing isolated CABG surgery.

## METHODS

This study was performed retrospectively on the perioperative data of 634 diabetic patients undergoing isolated CABG surgery in a single-center between September 2009 and January 2014. Of the patients, 65.1% (n=413) were male and 34.9% (n=221) were female. The average age was 60.5±9.1 years. After the local ethical committee approval, the data of the patients were collected from the archive records, hospital data recording program, patients' discharge summaries, operative reports, laboratory results, and radiological images. In this study, the relation between preoperative serum albumin levels and postoperative AKI development was observed. The AKI developing and non-AKI groups were compared.

AKI was diagnosed and evaluated according to the Kidney Disease: Improving Global Outcomes (KDIGO) classification^[9]^. The stages of AKI based on KDIGO classification are:

Stage 1: Increase in serum creatinine (SCr) ≥ 0.3 mg/dL (in 48 hours) or 1.5 to 1.9 mg/dL multiplied by baseline SCr (in seven days);

Stage 2: Between 2.0 to 2.9 mg/dL multiplied by baseline SCr;

Stage 3: 3.0 mg/dL or more multiplied by baseline SCr; increase in SCr ≥ 4.0 mg/dL; or beginning of renal replacement therapy regardless of a previous KDIGO stage.

Preoperative serum albumin levels were measured by the bromocresol green dye-binding method. The groups were not identified due to reference ranges but AKI development of patients regarding to serum albumin levels was observed.

Patients with chronic renal failure requiring hemodialysis or with SCr >1,6 mg/dL were excluded from this study^[9]^. And patients excluded from this study had systemic disorders associated with hypoalbuminemia, which were liver dysfunction, malnourishment, congestive heart failure, active malignancy, endocrinologic disorders (hypothyroidism, hyperthyroidism, etc.), lymphoproliferative disease, low hemoglobin (Hgb) levels (≤10 g/dL), active infection, and active or chronic autoimmune disease; patients taking steroids or chemotherapeutic drugs were also excluded.

Demographic characteristics and preoperative clinical conditions, like age, sex, body surface area, hypertension (HT) incidence, DM incidence previous myocardial infarction (MI), and preoperative ejection fraction (EF), were noted. Preoperative and postoperative laboratory results of creatinine, C-reactive protein (CRP), and Hgb were also noted. Perioperative data of cardiopulmonary bypass (CPB) and aortic cross-clamping (ACC) duration, postoperative drainage level, intubation duration, and ICU and in-hospital stays duration were measured for every individual patient. Proteinuria was measured by calorimetry.

In this study, HT was defined as a history of antihypertensive drug intake or blood pressure measurement ≥140/90 mmHg. In addition, DM was defined as history of antidiabetic drug intake or a measured fasting blood glucose level >126 mg/dL^[10]^. Peripheric venous blood of 5 to 7 cc was drawn to ethylenediaminetetraacetic acid (EDTA) vacutainers to prevent clotting from all the patients prior to surgery. All the hemogram parameters were measured in automatic Abbott CELL-DYN 3700 (Abbott Laboratory, Abbott Park, Illinois, USA) analyzers.

### Blood Glucose Level Measurements

Throughout the operation, blood glucose levels of the patients were evaluated conservatively, once before the CPB and then at hourly intervals with blood gas measurements. Crystallized insulin (Humulin R(r), Lilly, Indianapolis, USA) was applied intravenously to control high blood glucose levels. In the ICU, all patients' blood glucose levels were regulated with an insulin infusion according to the Portland protocol^[11]^. The total of the measured values was divided by the number of measurements to determine the mean blood glucose value during the operation for each patient. All surgical procedures were performed according to on-pump modern principles during CABG surgery. During the operation, following the routine application of anaesthesia, a median sternotomy was applied. A non-pulsatile roller pump and membrane oxygenator was used for CPB in all patients. The surgical procedure was performed at moderate systemic hypothermia (28°C-30°C). CPB was applied in a manner so that the flow rate was 2.2-2.5 L/min/m^2^, mean perfusion pressure was 50-80 mmHg, and hematocrit values were 20%-25%.

### Statistics

Statistical analysis was performed using the Statistical Package for Social Sciences (SPSS) software (SPSS version 21.0, IBM, Armonk, New York). Continuous variables with normal distribution were presented as mean (standard deviation [SD]), non-normal variables were reported as median (interquartile range [IQR]), and categorical variables were reported as percentage. Univariate comparisons between groups were performed using the chi-square test for categorical variables and the Student's t-test or Mann-Whitney rank sum test for continuous variables, as appropriate. Variables with a P-value <0.05 in univariate analysis were assessed in the multiple logistic regression model to determine the independent predictors of postoperative AKI. Receiver operating characteristic (ROC) curves were plotted to determine the optimal cut-off values for individual parameters in order to predict AKI and to establish the optimal cut-off points for use in clinical decision making. One-way repeated measure analysis of variance (ANOVA) was used to determine the change for creatinine over the first three postoperative days. A *P*-value <0.05 was considered to be significant.

## RESULTS

We included a total of 634 diabetic patients (60.59.1 years, 65.1% male) into this study. Eighty-three of them required coronary endarterectomy procedures with CABG and 13 underwent cardiac reoperation. The median ACC time was 51 (33-74) minutes. Mean baseline creatinine level was 1.05±0.60 (ranged from 0.4 to 6.6) mg/dL and creatinine level at the 48^th^ hour was 1.17±0.76 (ranged from 0.3 to 6.6) mg/dL. Two hundred and thirty (36.3%) patients developed AKI according to KDIGO classification. The preoperative mean blood urea level of the non-AKI group was 21.4±9.9 mg/dL and of the AKI group was 25.1±12.6 mg/dL (*P*<0,05). The postoperative mean blood urea level of the non-AKI group was 20.9±7.9 mg/dL and of the AKI group was 30.2±13.7 mg/dL (*P*<0.05). Preoperative, intraoperative, and postoperative clinical characteristics of AKI and non-AKI patients were summarized in Table 1.

**Table 1:** Baseline characteristics of the study subjects.

		AKI (n=230)	Non-AKI (n=404)	*P*-value
Preoperative data	Male % (N)	58.6 (135)	68.8 (278)	0.010
	Age (years)	61.8±9.1	59.7±9	0.005
	BMI	30.5±5.7	29.2±4.8	0.007
	Hypertension	51.7 (209)	41.8 (138)	<0.001
	Previous MI	7.4 (17)	7.6 (31)	0.495
	Previous cardiac surgery	1.3 (3)	2.5 (10)	0.379
	Fasting blood glucose (mg/dl)	182±59	171±54	0.023
	Proteinuria (mg)	15.2 (35)	12.5 (38)	0.004
	Creatinine (mg/dL)	1.2±0.89	0.95±0.33	<0.001
	Hemoglobin (g/dL)	12.2±1.7	13.0±1.7	<0.001
	RDW (%)	15.1±1.7	14.5±1.7	<0.001
	Albumin (g/dL)	3.96±0.47	4.1±0.48	<0.001
	Uric acid (mg/dL)	5.9±2	5.3±1.7	0.002
	C-reactive protein (mg/L)	1.54±2	1.61±2.9	0.742
	LVEF (%)	52.6±12.3	53.9±11.6	0.267
Intra-operative data	Drainage (mL)	550 (350-800)	600 (450-800)	0.317
	Intubation time (hour)	13 (10-19)	10 (8-13)	<0.001
	ACC time (minutes)	54 (32-82)	48.5 (33-70)	0.024
	CPB	89.5	87.3	0.412
Post-operative data	ICU stay (days)	70 (39-121)	55 (24-71)	<0.001
	Creatinine, first day (mg/dL)	1.6±1	0.95±0.31	<0.001
	Creatinine, second day (mg/dL)	1.67±1	0.88±0.30	<0.001
	Creatinine, third day (mg/dL)	1.71±1.2	0.90±0.34	<0.001

ACC=aortic cross-clamping; AKI=acute kidney injury; BMI=body mass index; CPB=cardiopulmonary bypass; ICU=intensive care unit; LVEF=left ventricular ejection fraction; MI=myocardial infarction; RDW=red cell distribution width

Female gender, older age, high body mass index (BMI) level, existence of HT, preoperative proteinuria, high baseline creatinine level, low Hgb, low albumin level, high uric acid level, and long ACC time were found to be related with high AKI development risk in univariate analysis. Multiple logistic regression analysis was used to determine the independent predictors of postoperative AKI development. High BMI levels (odds ratio [OR] and 95% confidence interval [CI], 1.066 [1.002-1.135]; *P*=0.043), existence of HT (OR and 95% CI, 2.153 [1.023-4.531]; *P*=0.043), existence of preoperative proteinuria (OR and 95% CI, 2.454 [1.007-5.984]; *P*=0.048), and low preoperative albumin levels (OR and 95% CI, 0.453 [0.216-0.947]; *P*=0.035) were found to be independent predictors for postoperative AKI development (Table 2).

**Table 2:** Multivariate predictors for acute kidney injury after coronary artery bypass grafting.

	Univariate OR, 95% CI	*P*-value	Multivariate OR, 95% CI	*P*-value
Male (N)	0.644(0.460-0.902)	0.010	0.572(0.288-1.137)	0.111
Age (years)	1.026(1.008-1.045)	0.005	1.006(0.971-1.043)	0.725
**BMI**	**1.046(1.012-1.082)**	**0.007**	**1.066(1.002-1.135)**	**0.043**
**Hypertension**	**2.195(1.434-3.360)**	**<0.001**	**2.153(1.023-4.531)**	**0.043**
**Proteinuria (mg)**	**2.094(1.260-3.480)**	**0.004**	**2.454(1.007-5.984)**	**0.048**
Preoperative Hgb (g/dL)	0.760(0.687-0.841)	<0.001	1.111(0.882-1.400)	0.370
Preoperative RDW (%)	1.218(1.106-1.342)	<0.001	1.045(0.862-1.266)	0.657
Baseline creatinine (mg/dL)	2.260(1.560-3.273)	<0.001	1.150(0.609-2.172)	0.665
Preop uric acid(mg/dL)	1.189(1.065-1.328)	0.002	1.086(0.918-1.284)	0.336
**Preoperative albumin (g/dL)**	**0.426(0.292-0.620)**	**<0.001**	**0.453(0.216-0.947)**	**0.035**
ACC time (minutes)	1.006(1.001-1.012)	0.024	1.001(0.993-1.009)	0.847

ACC=aortic cross-clamping; BMI=body mass index; CI=confidence interval; Hgb=hemoglobin; OR=odds ratio; RDW=red cell distribution width

**Fig. 1:**
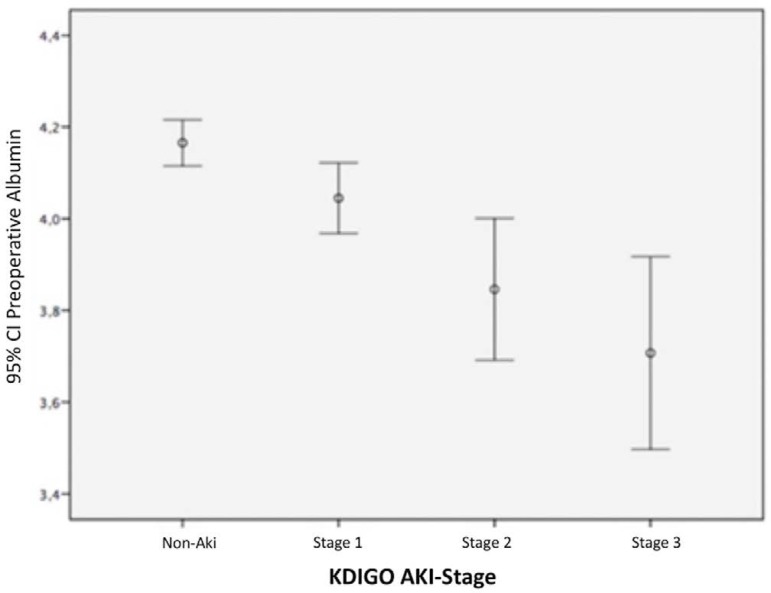
Preoperative serum albumin according to the acute kidney injury (AKI) stage. CI=confidence interval; KDIGO=Kidney Disease: Improving Global Outcomes

AKI developed patients had statistically significant lower serum albumin levels but higher rates of HT existence, proteinuria, and higher BMI levels than non-AKI patients (Table 1, Figure 1).

**Fig. 2:**
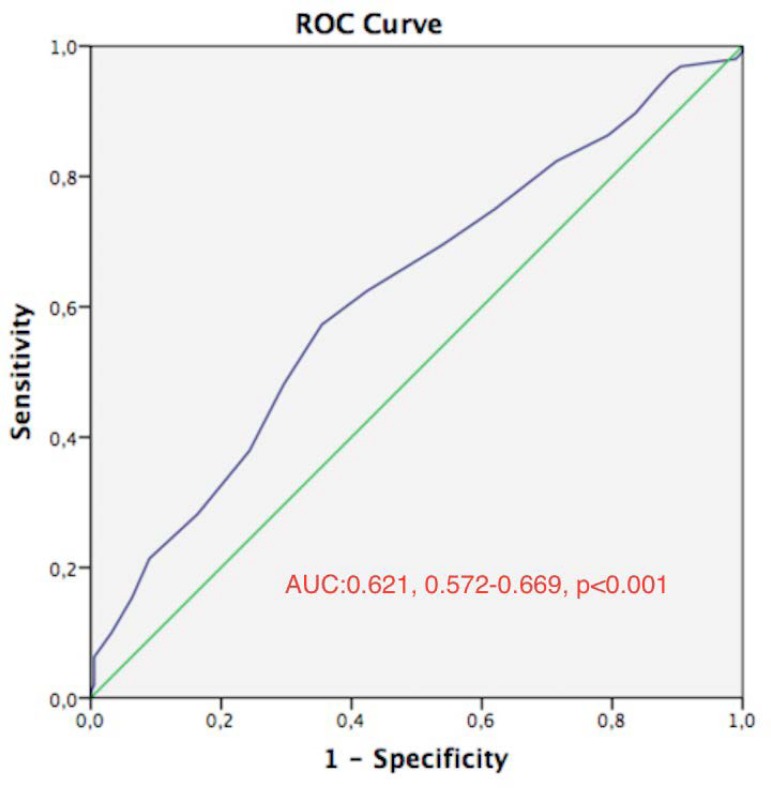
Receiver operating characteristic (ROC) curve plot for preoperative serum albumin in prediction of acute kidney injury (AKI). AUC=area under the curve

The diagnostic performance analysis made for each independent predictor showed that HT existence results in AKI development with a sensitivity of 78.8% and a specificity of 37% as proteinuria leads to AKI development with a sensitivity of 23% and a specificity of 88.3%. ROC curve analyses showed that albumin levels <3 mg/dL have 83% sensitivity and 10% specificity for predicting AKI development while BMI >29 has a 57% sensitivity and 53% specificity (Figure 2).

**Fig. 3:**
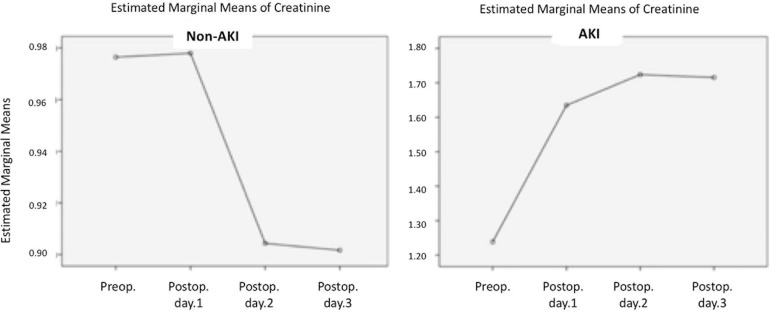
Estimated marginal means of creatinine. AKI=acute kidney injury

A one way-repeated measures ANOVA was conducted to determine the SCr level over the first three postoperative days in AKI and non-AKI groups. The assumption of sphericity was violated (Mauchly's test *P* value <0.001). There was a statistically significant change in creatinine level over three days in both groups. A significant increase in creatinine levels on postoperative first, second, and third days was observed in the AKI group (Figure 3).

In the overall population, the incidence of all-cause mortality was 8.2% (n=52) and of postoperative infection was 11% (n=70). Non-AKI patients were associated with less all-cause mortality (17.4% *vs*. 3.0%, *P*<0.001) and postoperative infection (16.5% *vs*. 7.9%, *P*=0.001).

## DISCUSSION

AKI following CPB is an important cause of morbidity and mortality^[12]^. In this study, our aim was to research the effect of preoperative low serum albumin levels on postoperative AKI development in diabetic patients undergoing isolated CABG operation.

AKI development subsequent to cardiac surgery is related with increased morbidity, mortality, and prolonged in-hospital stay. The incidence of AKI is 5%-30% after cardiac surgical procedures. Renal replacement therapy due to AKI development is the independent risk factor of mortality^[4,13]^. AKI following cardiac surgery is multi-factorial. The known risk factors are old age, diabetes, low preoperative glomerular filtration rate (GFR) (<60 mL/min/m^2^), low EF (<35%), and administration of nephrotoxic agents. The incidence of AKI was found to be high in patients with DM^[14-16]^. Our results showed that preoperative low serum albumin level, high BMI, and preoperative severe HT are related with enhanced risk of AKI development defined by KDIGO^[9]^ criteria.

Albumin is the primary protein to maintain the plasma oncotic pressure as it obtains 70% of the oncotic pressure^[17]^. It's an anti-inflammatory, antioxidant, and anticoagulant protein. Hypoalbuminemia is an indicator for liver and renal insufficiency. And hypoalbuminemia was found to be associated with increased mortality and morbidity subsequent to various surgical procedures in many studies^[6]^. Many studies have shown albumin not only to be an inflammatory marker but also an AKI predictor. Also, hypoalbuminemia is a strong predictor for end-stage renal failure. Albumin protects renal function by increasing the oncotic pressure in coronary artery disease patients, it provides the continuation of renal perfusion and improves the glomerular filtration, and it protects the kidneys from toxic agents. The effect of hypoalbuminemia on postoperative renal failure was shown in many studies^[6,18,19]^. Foley et al.^[20]^ showed that there is a strong correlation between hypoalbuminemia and ischemic heart disease. The same study revealed the need for hemodialysis in patients with low albumin levels in the same group of patients^[20]^. Wiedermann et al.^[21]^ showed hypoalbuminemia as an independent risk factor for AKI.

In the presented studies, it is shown that low serum albumin levels enhance the incidence of AKI development in patients who underwent CABG surgical procedure. Lee et al.^[22]^ performed a single-center, randomized, double blind trial with patients whose preoperative albumin level was <4mg/dL. They found out that a decreasing in serum albumin levels was associated with AKI. Fındık et al.^[23]^ have searched the relation between AKI and CABG procedure and have shown that patients with albumin levels <3.5 g/dL are tend to develop AKI more often. Patients with diabetes who underwent CABG procedure in our centre were isolatelly-maintained and had their details observed in this study. Serum albumin levels <3 g/dL are the independent risk factor for AKI development in diabetic patients who underwent CABG surgical procedure.

Engelman et al. found low BMI and low serum albumin levels associated with high postoperative mortality and morbidity in a study with 5168 CABG patients. The same study revealed that a low serum albumin level (<2.5g/dL) is an independent risk factor for postoperative bleeding, prolonged ICU stay, prolonged mechanical ventilation, and renal failure. This study also showed that a high BMI is related with increased sternum and saphenous vein wound infection risk^[7]^. However, our study found out that a high BMI is an independent risk factor of postoperative AKI development.

Wu et al.^[24]^ found preoperative proteinuria related with AKI development and as an independent risk factor for end-stage renal failure after CABG surgery. Hsu et al.^[25]^ evaluated the data of 600.000 patients and revealed that proteinuria and decrease in GFR are the independent risk factors for AKI development. Similarly, our study also found proteinuria as an independent risk factor for postoperative AKI development. In our study, high BMI, history of HT, and proteinuria were not found associated with AKI stage, except for the relation between presence of proteinuria and stage-3 AKI.

## CONCLUSION

We showed that preoperative low serum albumin level, high BMI, preoperative HT, and proteinuria are associated with AKI development, defined by the KDIGO classification, in the diabetic patients who underwent CABG surgical procedure postoperatively.

Serum albumin level <3 g/dL is an independent risk factor for AKI development in the isolated diabetic patients who underwent CABG surgical procedure. We emphasize that this adjustable albumin level should be considered before the operation since it is an easy and clinically implementable management for the prevention of AKI development.

**Table:** 

Authors' roles & responsibilities	
RA	Substantial contributions to the conception or design of the work; or the acquisition, analysis, or interpretation of data for the work; final approval of the version to be published
TA	Substantial contributions to the conception or design of the work; or the acquisition, analysis, or interpretation of data for the work; final approval of the version to be published
EY	Substantial contributions to the conception or design of the work; or the acquisition, analysis, or interpretation of data for the work; final approval of the version to be published
DC	Substantial contributions to the conception or design of the work; or the acquisition, analysis, or interpretation of data for the work; final approval of the version to be published
MS	Substantial contributions to the conception or design of the work; or the acquisition, analysis, or interpretation of data for the work; final approval of the version to be published
CK	Substantial contributions to the conception or design of the work; or the acquisition, analysis, or interpretation of data for the work; final approval of the version to be published
MBR	Substantial contributions to the conception or design of the work; or the acquisition, analysis, or interpretation of data for the work; final approval of the version to be published
